# Comparison of retrograde anatomy iliac bone flap grafting versus anterograde anatomy iliac bone flap grafting for treatment of osteonecrosis of the femoral head

**DOI:** 10.1186/s13018-023-03617-8

**Published:** 2023-02-22

**Authors:** Panfeng Wu, Yu Xiao, Liming Qing, Juyu Tang, Chengxiong Huang, Zheming Cao

**Affiliations:** Department of Orthopedics, Xiangya Hospital, Central South University, 87 Xiangya Road, Changsha, 410008 Hunan China

**Keywords:** Comparative study, Deep circumflex iliac artery, Iliac bone flap, Transplant, Osteonecrosis of the femoral head

## Abstract

**Background:**

Iliac bone flap with deep circumflex iliac artery is a common option in the treatment of Osteonecrosis of the femoral head (ONFH), and dissection of iliac bone flap is the key step for successful operation. This paper aims to introduce a new operative technique for dissecting iliac bone flap with deep circumflex iliac artery based on analysis of its advantages.

**Methods:**

A total of 49 patients treated by retrograde anatomy and 52 patients treated by anterograde anatomy from January 2010 to December 2020 were recruited. The two groups were then compared in terms of the preoperative baseline conditions, intraoperative data, and postoperative Harris hip score (HHS).

**Results:**

Compared with the retrograde anatomy group, the anterograde anatomy group had a significantly longer operating time, a significantly heavier intraoperative blood loss, a significantly higher rate of donor complication morbidity, a significantly higher rate of donor–recipient delayed healing, a significantly higher failure rate of iliac bone flap resection, a significantly higher rate of lateral femoral cutaneous nerve (LFCN) injury, and a significantly higher rate of ectopic ossification. No difference was found in postoperative HHS score between the two groups.

**Conclusion:**

As a new operative technique that can accurately locate the nutrient vessels of the iliac bone flap and quickly dissect the iliac bone flap with deep circumflex iliac artery while maintaining a comparable clinical effect, retrograde anatomy exhibited distinct advantages over anterograde anatomy in terms of simpler intraoperative operation, safer dissection, shorter operation time, lower blood loss, and fewer donor complications.

**Level of evidence:**

III, Retrospective.

**Supplementary Information:**

The online version contains supplementary material available at 10.1186/s13018-023-03617-8.

## Introduction

Osteonecrosis of the femoral head (ONFH), previously known as ischemic necrosis of the femoral head, is a common and refractory orthopedic disease worldwide. It was reported that around 20,000 new cases of osteonecrosis were diagnosed in the USA each year, and the cumulative number of patients with ONFH was 300,000 to 600,000 [1]. The first large-scale epidemiological survey of nontraumatic osteonecrosis in China showed that the estimated cumulative number of patients with nontraumatic ONFH had reached 8.12 million [2].

For patients with early femoral head necrosis, core decompression is a better treatment [3, 4]. When arterial ischemia is indicated by DSA and MRI results (stage 2B–3B of ARCO staging), vascularized transplantation will become a suitable option [5]. Vascularized grafting can repair the shape of the femoral head and restore blood flow in the necrotic area [6], thereby effectively controlling the progression of the disease and avoiding or delaying joint replacement. Common donor sites for vascularized grafting include the fibula flap and iliac crest flap [7–12]. Although free fibular graft is effective in restoring blood supply of the femoral head and providing mechanical support, it is associated with defects like trauma to the femoral neck and trochanter, mismatch between the donor and recipient vessels, symptomatic flexor hallucis longus contracture, and ankle pain [7, 13–15]. The iliac crest bone structure is rich in cancellous bone that is conducive to healing, and can thereby promote postoperative recovery. Besides, it also has the advantages of sufficient blood supply, easiness to cut, and ability to be trimmed at will [16]. Out of comprehensive considerations, the free iliac bone flap is generally preferred for the treatment of ONFH in our center.

Due to the anatomical advantages of the iliac bone flap, it is widely used in the reconstruction and repair of the whole body [17–20]. With the development of microsurgical techniques, iliac bone flap transplantation with anterolateral anatomy of deep circumflex iliac vessels was proposed to repair femoral head necrosis [21]. The current clinical method of iliac bone flap dissection is to directly cut into the level of the internal oblique muscle at the body surface projection point of the deep circumflex iliac artery to locate and dissect it [22]. In view of a direct search for the deep circumflex iliac artery, this method is referred to as anterograde anatomy. In clinical applications, we found that accurate positioning of the deep circumflex iliac artery could not be achieved in some cases, which resulted in a prolonged operation time, a high incidence of postoperative complications, and a risk of surgical failure. In order to overcome these problems, we plan to explore a new iliac crest flap operative technique that can effectively improve the success rate, and to promote this technique in clinical practice.

## Materials and methods

### Patients

A total of 101 patients with ONFH who accepted treatment at the Central South University of Xiangya Hospital from January 2010 to December 2020 were recruited. Specifically, 49 patients were treated with retrograde anatomy, and 52 patients were treated with anterograde anatomy. The osteonecrosis was diagnosed and classified for stages on the basis of plain radiographs and magnetic resonance images according to the ARCO classification system.

The inclusion criteria were: (1) Symptomatic ARCO stage II to III osteonecrosis, (2) Failure of conservative treatment, (3) Aged ≤ 55 years, (4) No history of hip infection or hip surgery, and (5) Completion of an HHS score at least 24 months postoperatively. This study was approved by the Institutional Review Board of the Central South University of Xiangya Hospital, and informed consent forms were obtained from all the patients included.

The anterograde anatomy group comprised 37 male and 15 female subjects, and the mean age at operation was 35.4 years. For BMI status, 41 subjects were normal and 11 were abnormal. For causes of osteonecrosis, 13 subjects were due to steroid use, 8 due to alcohol abuse, 25 due to post-traumatic status, and 6 due to idiopathic disease. Per radiographic evaluation, 40 hips were graded as ARCO stage II, and 12 hips graded as stage III. The retrograde anatomy group comprised 31 male and 18 female subjects, and the mean age at operation was 34.0 years. For BMI status, 35 subjects were normal and 14 were abnormal. For causes of osteonecrosis, 12 subjects were due to steroid use, 11 due to alcohol abuse, 21 due to post-traumatic status, and 5 due to idiopathic disease. Per radiographic evaluation, 36 hips were graded as ARCO stage II, and 13 hips graded as stage III (Table 1).

**Table 1:** Demographic data

Variable	Retrograde group	Antegrade group	*P* value^#^
	(*N* = 49)	(*N* = 52)	
Age (year)	34.0 ± 8.1	35.4 ± 9.3	0.417
Sex			0.525
Male	31	37	
Female	18	15	
BMI			0.49
Normal	35	41	
Abnormal	14	11	
Alcohol history			1
Yes	18	19	
No	31	33	
Smoking history			0.547
Yes	21	18	
No	28	34	
Chronic diseases*			0.797
Yes	8	10	
No	41	42	
Etiology			0.834
Glucocorticoid	12	13	
Alcoholic	11	8	
Traumatic	21	25	
Idiopathic	5	6	
ARCO stage			0.818
II	36	40	
III	13	12	

^#^Two-sided Fisher’s exact test

^*^Hypertension, diabetes, coronary heart disease, etc.

### Operative technique

**Fig. 1:**
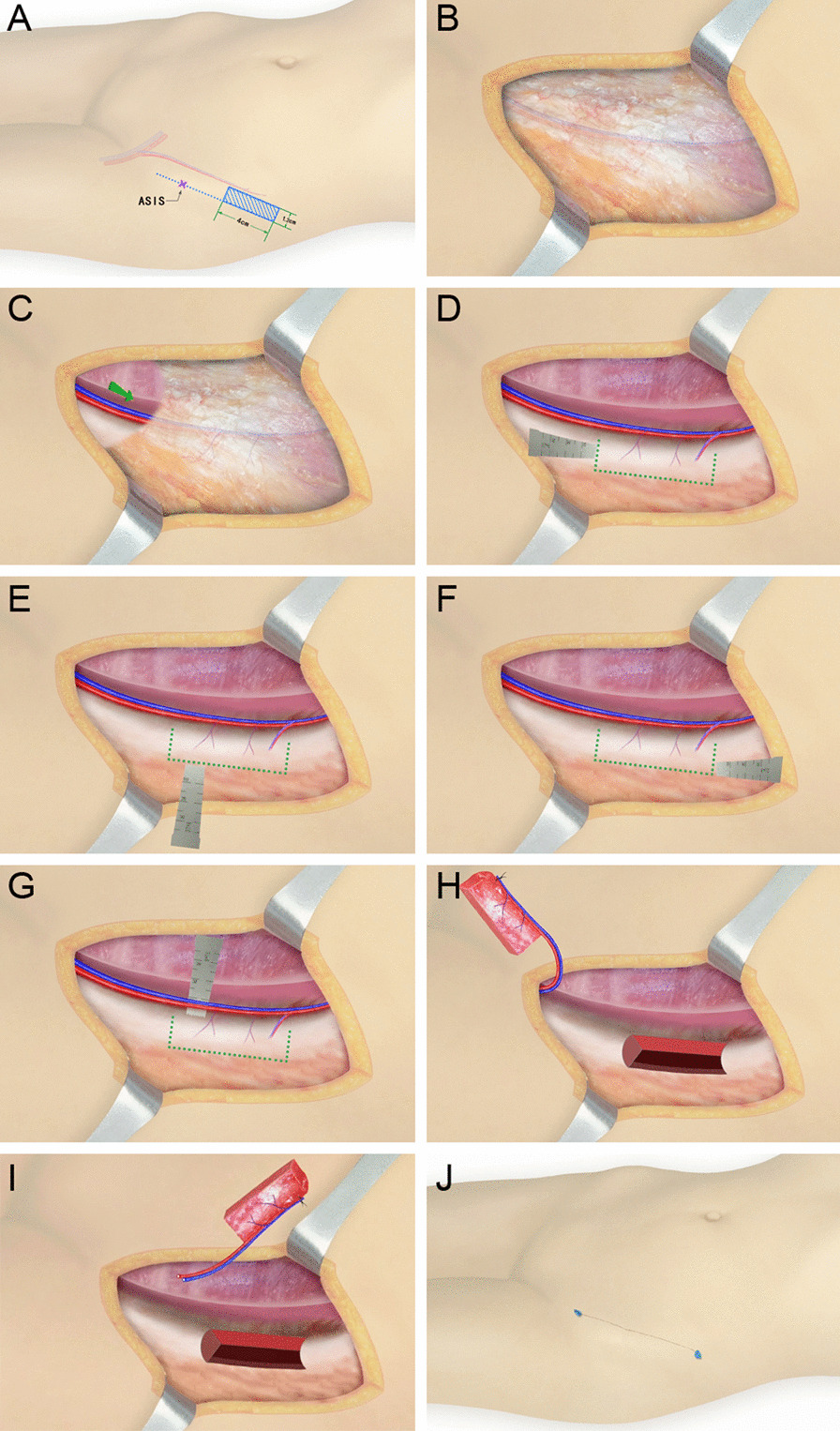
Schematic diagram of iliac bone flap with deep circumflex iliac artery cut by "anterograde anatomy." **A** Preoperative design of iliac bone flap resection. **B** The aponeurosis of the external oblique was exposed. **C** The internal oblique muscle was cut at the midpoint of the inguinal ligament to find the main shaft of the deep circumflex iliac vessel, which was then followed up the main shaft until the iliac bone flap and perforator were completely exposed. **D**–**G** Bone knife chiseling iliac bone flap. **H**–**I** Complete free iliac bone flap. **J** The supply area is directly closed

**Fig. 2:**
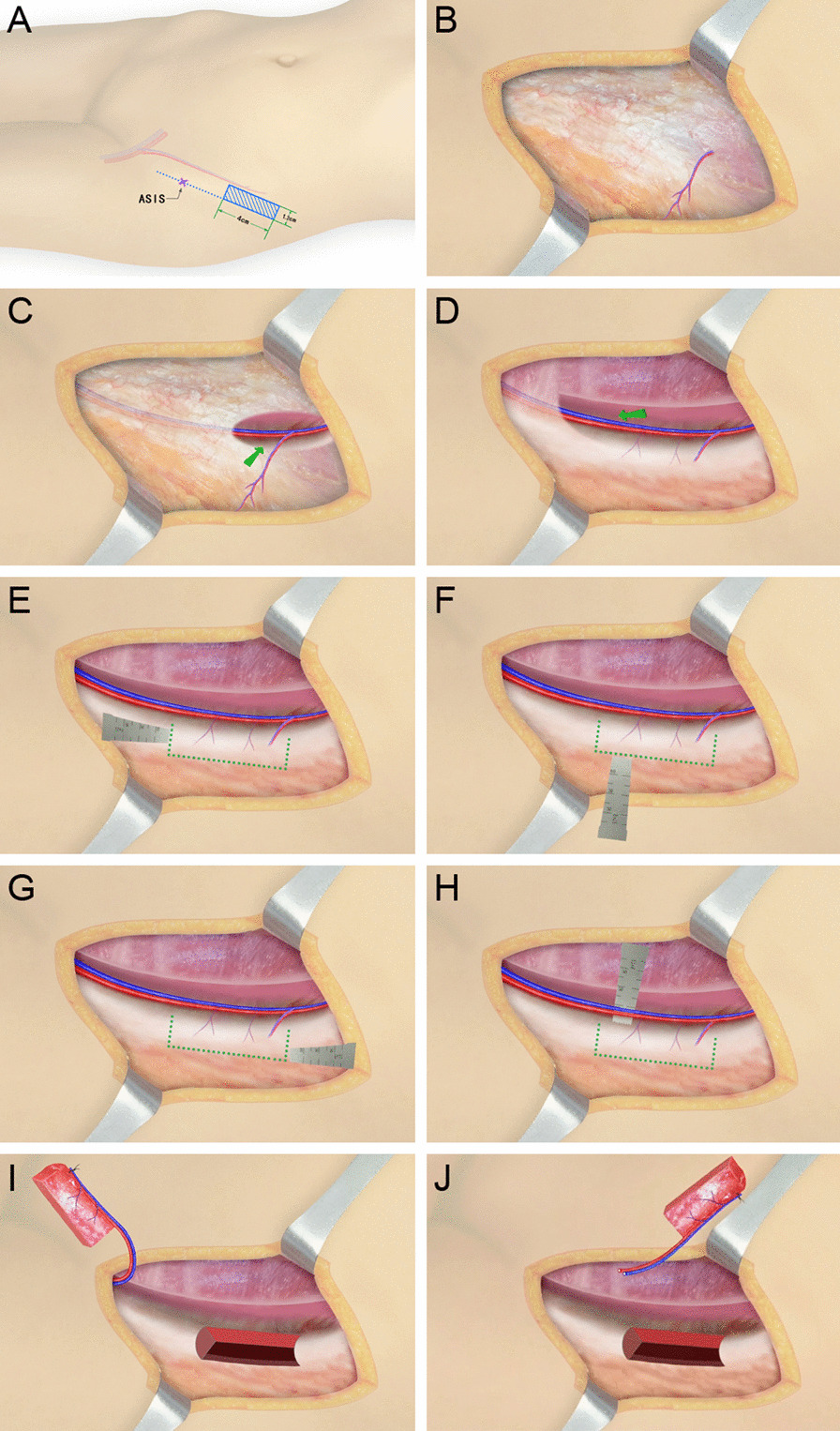
Schematic diagram of iliac bone flap with deep circumflex iliac artery cut by "retrograde anatomy." **A** Preoperative design of iliac bone flap resection. **B** Look for suitable cutaneous perforators near the iliac tubercle and the medial side of the iliac bone, and then the cutaneous perforator or muscular branch separated from the surface to the interior. **C** Find the main trunk of deep iliac circumflex artery on the deep surface of internal oblique muscle. **D** Separate the deep circumflex iliac artery to its origin. **E**–**H** Bone knife chiseling iliac bone flap. **I**, **J** Complete free iliac bone flap

**Fig. 3:**
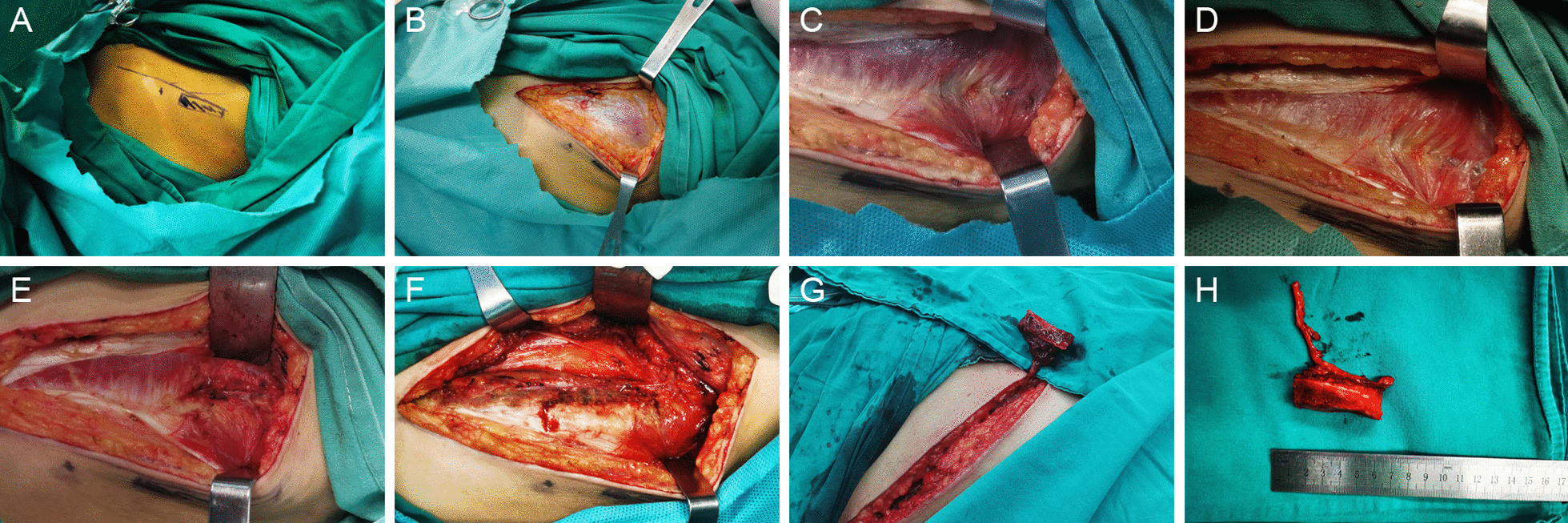
Surgery demonstration of retrograde anatomy for iliac bone flap with deep circumflex iliac artery. **A** Preoperative design of iliac bone flap resection. **B**, **D** The skin and subcutaneous tissue were dissected to expose the aponeurosis of obliquus externus abdominis and the perforator of the deep circumflex iliac vessel. **E**, **F** Retrograde dissection along the perforating branch to the deep surface of the internal oblique muscle; the trunk of the deep circumflex iliac vessels was exposed; **G** the iliac bone flap and the deep circumflex iliac vessels were completely isolated. **H** Harvested the iliac bone flap with trunk of deep iliac circumflex vessel

All the operations were performed by the same surgery team from the Department of Hand and Microsurgery. The procedure of anterograde anatomy has been described in detail in one of our published articles (Fig. 1, Additional file 1: Video 1) [21]. The iliac bone flap with deep circumflex iliac blood vessel was cut by the retrograde dissection method. Firstly, the skin and subcutaneous tissue were dissected along 1 cm inside the anterior superior iliac spine parallel to the inguinal ligament and iliac crest. The superficial fascia tissue was cut by a needle-type electrosurgical knife to expose the aponeurosis of obliquus externus abdominis to search for the appropriate musculocutaneous perforator near the iliac tubercle and inner side of the iliac bone. If no appropriate musculocutaneous perforator was found, the obliquus externus abdominis would be cut parallel to the skin incision to search for the musculocutaneous perforator near the iliac tubercle, inside the iliac bone, and on the surface of the obliquus internus abdominis. The musculocutaneous perforator was then dissected from the surface to the inside with microscissors and mosquitoclamp under a surgical magnifying glass. The medial branch of the iliac crest within the obliquus internus abdominis was exposed to determine the direction of blood vessel source and dissect the vessel by reverse its course. The obliquus internus abdominis was cut by the needle-type electrosurgical knife while retaining a thin layer of muscle fibers on the surface of the blood vessels. The muscle fibers were cut by microscissors, protected the branches entering the inner plate of the iliac crest, and ligatured the branches emanating from the medial side of the vessels. The lateral femoral cutaneous nerve could be found crossing diagonally with the vessels near the anterior superior iliac spine and below the medial side of the vessels. While the iliac bone flap was harvested, the lateral femoral cutaneous nerve was carefully protected by elaborative disassociation between the nerve and its surrounding tissues. Then, the deep circumflex iliac artery was sharp-dissected from its intersection with the lateral femoral cutaneous nerve to its origin of the iliac inguinal area (for the convenience of cutting the pedicle of a suitable caliber and length). Finally, the iliac bone flap with trunk of deep circumflex iliac blood vessel was excised (Figs. 2, 3, Additional file 2: Video 2). Blood halo of the bone flap was examined before cutting the perforating vessel pedicle in both groups to ensure that no perforating vessel was damaged in the process of cutting (Additional file 3: Video 3).

The intraoperative and postoperative outcomes of the two groups were recorded to determine which surgical protocol was superior. Follow-up examinations were performed 3, 6, 9, 12, 18, and 24 months after discharge. All the hips were evaluated based on the HHS system [23]. Radiographs and computed tomography/magnetic resonance images were used to assess the viability of the bone flap, the healing of necrotic lesion, and the evidence of postoperative necrotic progression [24]. The surgical outcome was rated as excellent if HHS ≥ 90, good if 80 < HHS < 89, fair if 70 < HHS < 79, and poor if HHS < 70. The clinical results including HHS, radiographic progression, and rate of excellent or good outcomes were compared between the two groups.

### Statistical analysis

The quantitative data were expressed as mean ± standard deviation, and the preoperative and postoperative data were compared by paired t-test. The comparisons among two or multiple independent samples were performed by the one-way ANOVA test. The qualitative data were expressed as numbers or percentages, and was compared using the *χ*^2^ test and Fisher’s exact test. Statistical analysis was performed by SPSS 20.0 software (SPSS Inc., US), and *P* < 0.05 was considered statistically significant.

## Results

A total of 101 subjects with ONFH were included in this preoperative, intraoperative, and postoperative study that compared retrograde anatomy versus anterograde anatomy. No significant differences were observed in terms of age, sex, BMI, alcohol history, smoking history, chronic diseases, etiology, ARCO stage, preoperative HHS scores, or bone flap dimensions between the retrograde anatomy and anterograde anatomy groups. The homogeneity of the two groups was good, and the intraoperative and postoperative results were both comparable (Tables 1, 2, 3).

**Table 2:** Intraoperative and postoperative data

Variable	Retrograde group	Antegrade group	*P* value
	(*N* = 49)	(*N* = 52)	
Iliac bone flap volume (cm^3^)	15.6 ± 5.9	15.5 ± 5.6	0.905
Total—operation time (min)	173.3 ± 16.5	202.1 ± 16.8	< 0.001
Iliac bone flap resection time (min)	44.1 ± 4.4	57.1 ± 7.6	< 0.001
Vascular anastomosis time (min)	27.8 ± 2.2	27.8 ± 2.3	0.948
Total—operative blood loss (ml)	327.5 ± 60.4	368.3 ± 44.3	< 0.001
Donor blood loss (ml)	115.3 ± 29.2	149.0 ± 28.8	< 0.001
Recipient blood loss (ml)	212.2 ± 38.9	219.2 ± 24.6	0.287
Donor complication	1 (2.0%)	11 (21.2%)	0.004
Infection	0	3	
Hematoma	1	8	
Recipient complication	1 (2.0%)	2 (3.8%)	1
Infection	0	0	
Hematoma	1	2	
Donor-recipient delayed healing	0 (0.0%)	6 (11.5%)	0.027
Failure of iliac bone flap resection^a^	2 (4.1%)	12 (23.1%)	0.008
Location of iliac bone flap incision			0.008
Contralateral	47	40	
Ipsilateral	2	12	
LFCNI	2 (4.1%)	11 (21.2%)	0.015
Recover within 3 months	2	6	
Recipient ectopic ossification	3 (6.1%)	15 (28.8%)	0.004
Location of iliac bone flap incision			0.442
Contralateral	1	2	
Ipsilateral	2	13	

*LFCNI* lateral femoral cutaneous nerve injury

^a^The patients who failed resection were replaced by ipsilateral iliac bone flap resection

**Table 3:** Harris hip function scale was compared between the two groups at preoperative and postoperative time points

Variable	Retrograde group	Antegrade group	*P* value
	(*N* = 49)	(*N* = 52)	
Preoperative surgery	65.9 ± 4.7	65.8 ± 5.0	0.888
Postoperative surgery			
9 months	73.4 ± 5.2	73.0 ± 5.2	0.562
12 months	78.0 ± 4.1	77.9 ± 4.6	0.928
18 months	81.3 ± 3.9	81.3 ± 4.0	0.986
24 months	83.0 ± 4.2	83.2 ± 4.0	0.77
36 months	84.6 ± 4.7	84.4 ± 4.3	0.855
RP	2 (4.1%)	3 (5.8%)	1
EGR	42/7 (85.7%)	43/9 (82.6%)	0.788

*RP* radiographic progression, *EGR* means excellent and good rate

Compared with the retrograde anatomy group, the anterograde anatomy group had a significantly longer operation time (202.1 ± 16.8 vs 173.3 ± 16.5 min, *P* < 0.001), a significantly heavier intraoperative blood loss (368.3 ± 44.3 vs 327.5 ± 60.4 ml, *P* < 0.001). There into, the iliac bone flap resection time of the anterograde anatomy group was significantly longer than that of the retrograde anatomy group (57.1 ± 7.6 vs 44.1 ± 4.4 min, *P* < 0.001). Similarly, the donor blood loss of the anterograde anatomy group was significantly heavier than that of the retrograde anatomy group (149.0 ± 28.8 vs 115.3 ± 29.2 ml, *P* < 0.001). No differences were found in recipient blood loss and vascular anastomosis time between the two groups. The donor complication morbidity rate in the anterograde anatomy group outnumbered that in the retrograde anatomy group (21.2% vs 2.0%, respectively, *P* = 0.004). Specifically, the anterograde anatomy group had 3 infection and 8 hematoma cases, while the retrograde anatomy group had no infection and only 1 hematoma case. The donor-recipient delayed healing rate in the anterograde anatomy group outnumbered that in the retrograde anatomy group (11.5% vs 0.0%, respectively, *P* = 0.027). A higher failure rate of iliac bone flap resection was observed in the anterograde anatomy group than in the retrograde anatomy (23.1% vs 4.1%, respectively, *P* = 0.008). A higher rate of LFCN injury was observed in the anterograde anatomy group than in the retrograde anatomy (21.2% vs 4.1%, respectively, *P* = 0.015). Within 3 months after operation, 6 patients with LFCN injury in the anterograde anatomy group and 2 patients with LFCN injury in the retrograde anatomy group recovered, and the injury was considered to be possible intraoperative tension nerve injury. The incidence of ectopic ossification after anterograde iliac flap resection was significantly higher than that in the retrograde iliac flap resection group (28.8% vs 6.1%, respectively, *P* = 0.004). Among these patients, there was no statistical difference in the number of cases between the ipsilateral and contralateral iliac bone flap group, and most of them occurred in the ipsilateral group (Table 2).

**Fig. 4:**
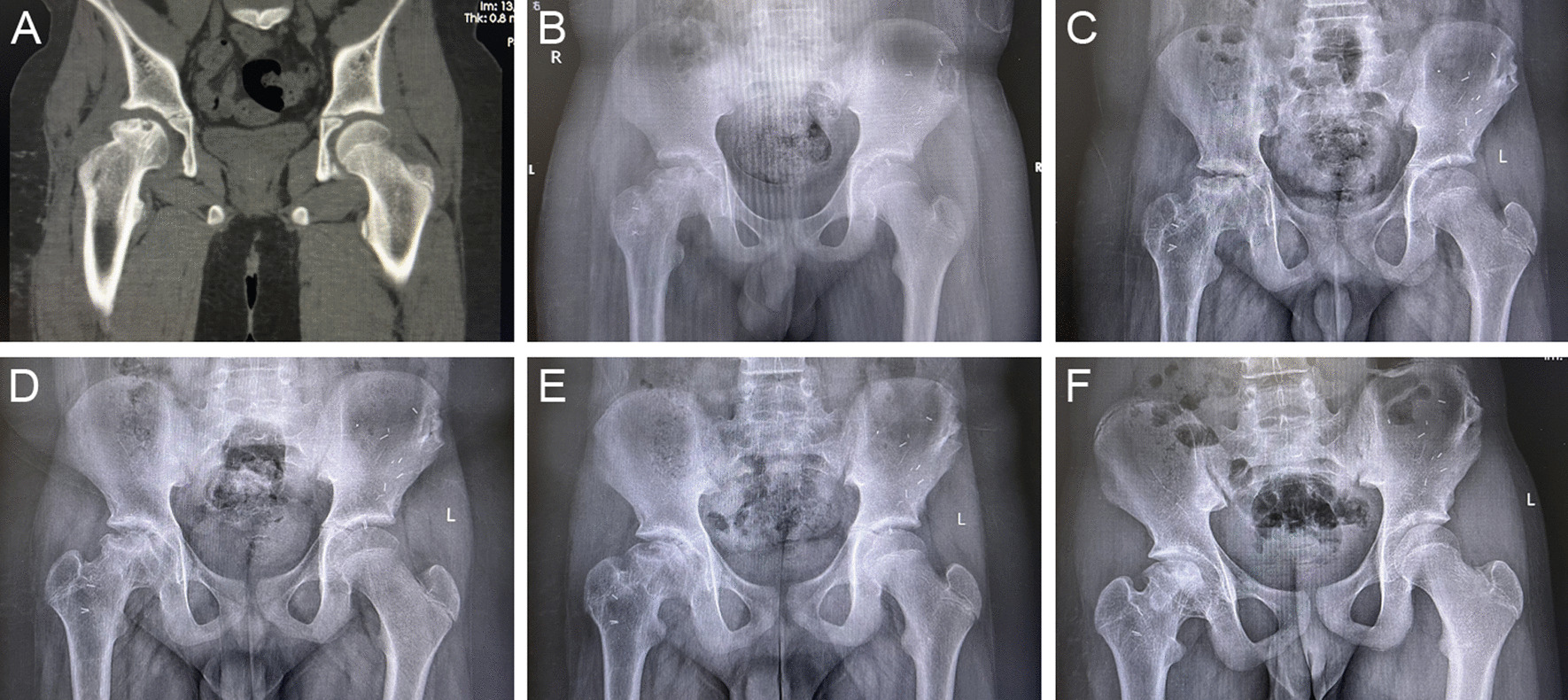
Case 1: A 11-year-old men patient with ARCO III traumatic ONFH at right received the treatment of anterograde anatomy iliac bone flap transfer. **A** Preoperative radiograph of CT image: note the distinct flattening, clear necrosis and osteolysis. **B**–**F** The anteroposterior radiograph of the pelvis at 1 months, 3 months, 6 months, 12 months, 24 months postoperatively: through the whole follow-up, round shape of femoral head is protected, with no progressive collapse and a well-preserved joint space. Iliac graft incorporates with the femoral head better with prolonged follow-up

**Fig. 5:**
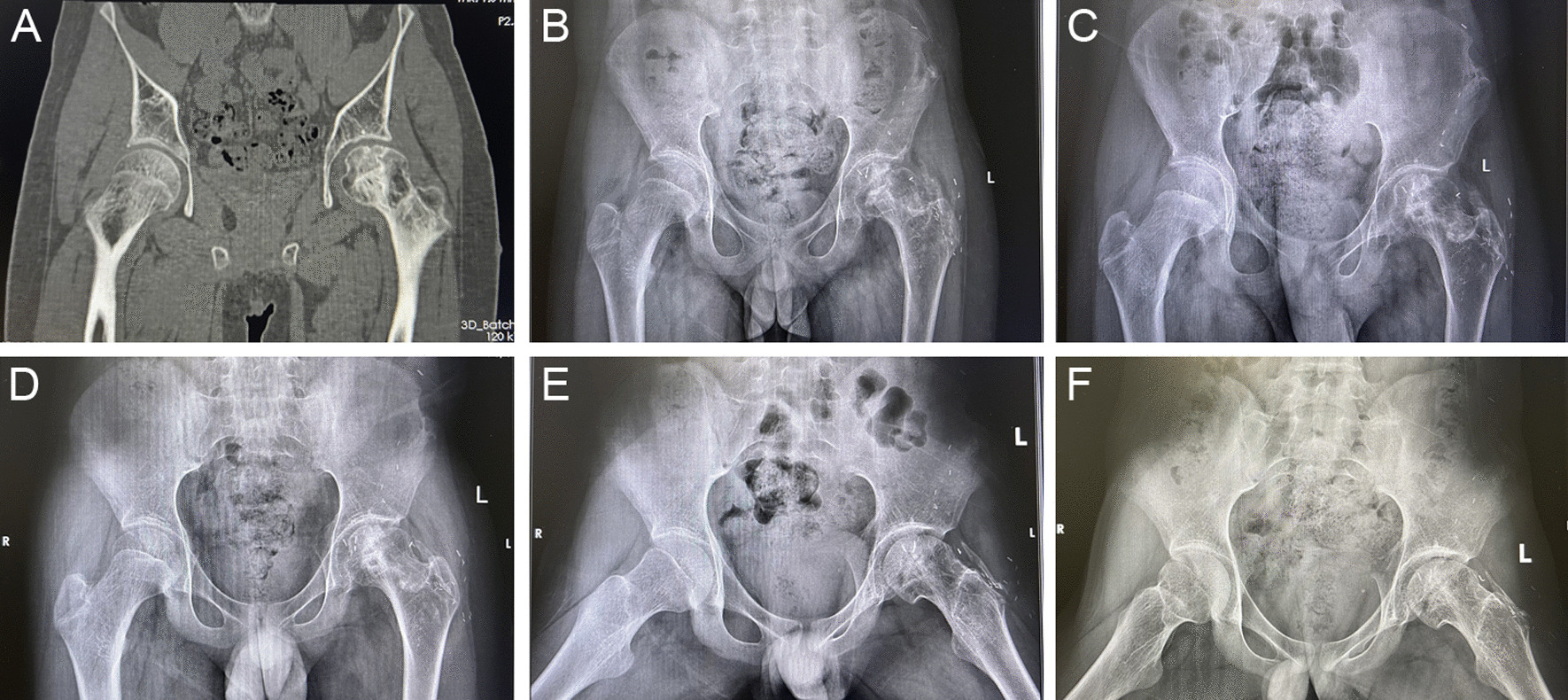
Case 1: A 12-year-old men patient with ARCO III traumatic ONFH at left received the treatment of retrograde anatomy iliac bone flap transfer. **A** Preoperative radiograph of CT image: the articular plane is uneven, there is obvious necrosis and osteolysis. **B**–**F** The anteroposterior radiograph of the pelvis at 1 months, 3 months, 6 months, 12 months, 24 months postoperatively: During the whole follow-up, the shape of the femoral head gradually became round without progressive collapse, and the joint space was well preserved

In radiographic evaluation, the bone graft was in good cohesion with the femoral head, and the joint space was well preserved. Specifically, 82.6% of the anterograde anatomy group and 85.7% of the retrograde anatomy group achieved excellent or good outcomes. Radiographic progression was observed in 3 patients in the anterograde anatomy group and 2 patients in the retrograde anatomy group. The change of postoperative HHS score showed no difference between the two groups at any follow-up point (Table 3, Figs. 4, 5).

## Discussions

ONFH, as a common intractable disease, is characterized by local death of osteocytes and the component of the bone marrow occurs owing to venous stasis or arterial blood supply damage or interruption in the femoral head. Vascular iliac bone flap transplantation can improve or reconstruct the blood supply in the femoral head, promote new bone formation, maintain the spherical shape of femoral head, and prevent the collapse of the femoral head, thereby improving clinical symptoms and delaying the demand for hip replacement [26–30]. However, postoperative pain, lateral femoral cutaneous nerve injury, hematoma formation and other problems still exist [31, 32]. Therefore, this research center began to gradually explore a new method of iliac bone flap resection to solve the above problems.

The results of this study showed that both retrograde anatomy and anterograde anatomy of iliac bone flap transplantation in the treatment of ONFH achieved good clinical efficacy. Iliac bone flap, as a sort of cancellous bone, is an excellent choice of autologous donor site. The cancellous bone is primarily osteoconductive, but is also osteoinductive and osteogenic, thus demonstrating all key properties [16]. Cancellous autograft can revascularize within several weeks and induce more bone formation than cortical graft, and will be replaced by host bone and marrow within 1 year [33–35]. Hence, the iliac bone flap can fuse well with the femoral head, promote revascularization, and provide a good shape and volume [19, 36]. If vascular anastomosis fails, as a traditional cortical bone graft, iliac bone is superior to other bone flaps [37]. Previous research results of our center have shown that anterograde resection of vascular iliac bone flap transplantation in the treatment of femoral head necrosis has achieved good clinical efficacy [21, 25]. Pedicled iliac bone flap transfer and anterograde iliac bone flap grafting exhibited identical clinical results with respect to arresting the exacerbation of osteonecrosis, and the degree of recovery was correlated with ARCO stage [25].

The decisive step in a successful operation of iliac bone flap for femoral head necrosis is the successful dissociation of the deep circumflex iliac artery. At present, anatomical studies have found that the anterior part of the iliac bone has multiple sources of blood supply and the deep circumflex iliac vessel is one of its main supply vessels [38–40]. The uncertainty of its source, course, type, caliber, and location raises a considerable challenge to intraoperative anatomy [41, 42], which may easily cause vascular injury, result in failure of operation, and aggravate the injury of abdominal muscles. The deep circumflex iliac artery has great variations in the course in the pelvis and origin [43]. In the iliac inguinal area, the external iliac artery or femoral artery can emit inferior epigastric artery, and superficial or deep circumflex iliac artery. The three vessels are located close to each other. During the operation of anterograde anatomy, it is difficult to determine whether these vessels can ultimately nourish the iliac bone flap [44]. The deep circumflex iliac artery clings to the inguinal ligament and runs outward to the anterior superior iliac spine, with one or two ascending branches reaching the abdominal wall and running between the internal oblique and transverse abdominis. Besides, it has been shown to give off some small branches at the plane of the iliac tubercle to nourish the iliac muscle and one or two musculocutaneous perforators to nourish the skin in this area [45]. Therefore, we plan to take advantage of this anatomical feature to accurately locate the deep circumflex iliac artery, the nutrient vessel of the iliac bone flap, through retrograde dissection with the musculocutaneous perforator at the plane of the iliac tubercle. This method can avoid wrong dissection of the deep circumflex iliac artery due to inaccurate judgment in anterograde anatomy.

Retrograde anatomy searches for the musculocutaneous perforator in the plane of the external oblique aponeurosis and then cuts the obliquus externus abdominis according to the course of it, thus avoiding serious injury of the external oblique muscle caused by traditional anterograde anatomy. In addition, retrograde anatomy only needs to ligate the musculocutaneous artery from the inner side of the vessels, while the branches from the outer side of the vessels that enter the inner plate to nourish the iliac bone are not interfered, so that intraoperative bleeding can be minimized. The whole procedure of retrograde anatomy is required to be carried out using microscissors and mosquitoclamp under microscope. In this way, the precision of surgical manipulation can be improved and the intraoperative bleeding, lateral femoral cutaneous nerve injury, and ectopic ossification can be effectively reduced. During the operation process, the suitable separation plane of the deep circumflex iliac artery can be selected according to the diameter of the vessels in the recipient area so as to improve the success rate of iliac bone flap transplantation. The cutting method of retrograde iliac bone flap is similar to the idea of "retrograde anatomical" perforator flap. In both cases, the main vessels of the graft are dissected after confirming the reliability of the perforator vessels of the graft (suitable for the required length and corresponding vessel diameter of the donor area) [46, 47]. For the above advantages, it can be considered that retrograde anatomical iliac bone flap transplantation technology is worthy of further exploration and promotion.

## Conclusions

Retrograde anatomy and anterograde anatomy exhibited identical clinical effects with respect to arresting the exacerbation of osteonecrosis, alleviating hip pain, and enhancing hip function for patients with pre-collapse ONFH. However, retrograde anatomy is featured by simpler intraoperative operation, safer dissection, shorter operation time, lower blood loss, and fewer donor complications. Thus, we consider that retrograde anatomy, as a novel operative technique for dissecting iliac bone flap with deep circumflex iliac artery, is more suitable for the treatment of ONFH.

## Supplementary Information

**Additional file 1: Video 1.** Animation of anterograde iliac bone flap removal procedure.

**Additional file 2: Video 2.** Animation of retrograde iliac bone flap removal procedure.

**Additional file 3: Video 3.** Blood flow of the iliac bone flap after dissection and before vessel dissection.

## Data Availability

The data sets to support the findings of this study are included within the article, including figure, tables, and supplementary figure. Any other data used to support the findings of this study are available from the corresponding author upon request.

## Abbreviations

ONFH: Osteonecrosis of the femoral head
FVIBF: Free vascularized iliac crest flap
BMI: Body mass index
DSA: Digital substraction angiography
MRI: Magnetic resonance diffusion tension imaging results
ARCO: Association research circulation osseous
LFCNI: Lateral femoral cutaneous nerve injury
RP: Radiographic progression
EGR: Means excellent and good rate
